# Maternal depressive symptoms during pregnancy are associated with amygdala hyperresponsivity in children

**DOI:** 10.1007/s00787-017-1015-x

**Published:** 2017-06-30

**Authors:** Noortje J. F. van der Knaap, Floris Klumpers, Hanan El Marroun, Sabine Mous, Dirk Schubert, Vincent Jaddoe, Albert Hofman, Judith R. Homberg, Henning Tiemeier, Tonya White, Guillén Fernández

**Affiliations:** 1 Department of Cognitive Neuroscience, Donders Institute for Brain, Cognition, and Behaviour, Radboud University Medical Centre, P.O. Box 9101, 6500 HB Nijmegen, The Netherlands; 2 Experimental Psychopathology and Treatment Section, Behavioural Science Institute, Radboud University Nijmegen, Nijmegen, The Netherlands; 3 Department of Child and Adolescent Psychiatry, Erasmus Medical Centre, 3000 CB Rotterdam, The Netherlands; 4 Department of Epidemiology, Erasmus Medical Centre, 3000 CB Rotterdam, The Netherlands; 5 Department of Epidemiology, Harvard T.H. Chan School of Public Health, Boston, MA USA; 6 Department of Radiology, Erasmus Medical Centre, 3000 CB Rotterdam, The Netherlands; 7 Department of Paediatrics, Erasmus Medical Centre, 3000 CB Rotterdam, The Netherlands

**Keywords:** Depression, Prenatal, Amygdala, fMRI, Child

## Abstract

Depression during pregnancy is highly prevalent and has a multitude of potential risks of the offspring. Among confirmed consequences is a higher risk of psychopathology. However, it is unknown how maternal depression may impact the child’s brain to mediate this vulnerability. Here we studied amygdala functioning, using task-based functional MRI, in children aged 6–9 years as a function of prenatal maternal depressive symptoms selected from a prospective population-based sample (The Generation R Study). We show that children exposed to clinically relevant maternal depressive symptoms during pregnancy (*N* = 19) have increased amygdala responses to negative emotional faces compared to control children (*N* = 20) [*F*(1,36) 7.02, *p* = 0.022]. Strikingly, postnatal maternal depressive symptoms, obtained at 3 years after birth, did not explain this relation. Our findings are in line with a model in which prenatal depressive symptoms of the mother are associated with amygdala hyperresponsivity in her offspring, which may represent a risk factor for later-life psychopathology.

## Introduction

In 7–15% of all pregnancies, the mother encounters depressive symptoms, which may increase some risks of her unborn child [1]. Indeed, children prenatally exposed to maternal depressive symptoms show more often prematurity, anxiety, impulsive behavior and sleep problems at young ages than non-exposed peers [2–4]. Moreover, as they mature, these children have more affective problems and antisocial behavior than children of euthymic mothers [3–5]. However, the mechanistic link between maternal depression during pregnancy and the child’s mental health is not well understood. The amygdala is a core structure in emotional processing [6, 7]. Altered amygdala function is thought to underlie affective symptoms and increased risk of psychopathology [4, 8–11]. A study on newborns exposed to maternal depression shows altered amygdala microstructure as represented by lower fractional anisotropy and axial diffusivity and altered functional connectivity of the amygdala [12, 13]. Initial studies on school-aged children revealed lasting differences in cortical thickness associated with exposure to prenatal maternal depression [14, 15]. While these studies might suggest lasting structural effects, it is currently unclear whether amygdala functionality is affected at older ages. Probing this question could provide further insight into the elevated risk of mental disorders, which typically becomes apparent later in life.

Thus, we conducted a functional magnetic resonance imaging (fMRI) study, using an emotional face matching task, in a prospective community sample of children aged 6–9 years [16]. We hypothesized that exposure to maternal depressive symptoms during pregnancy is associated with amygdala hyperresponsivity for emotional faces in school-aged children.

## Methods

### Participants

Subjects took part in ‘The Generation R Study’, a prospective population-based birth cohort study investigating the health and development of children in the Netherlands [17]. At the age of 6 years, a brain MRI study began within a subsample of the Generation R participants. This pilot contained an MRI session with a structural scan, diffusion tensor imaging and resting state functional imaging. A total of 1070 children were scanned focusing on studies investigating prenatal exposure to potentially harmful agents (e.g., nicotine, maternal depression or alcohol), or specific behavioral problems (e.g., ADHD or antisocial behavior). fMRI tasks were added to the imaging pipeline for specific studies whenever possible. Within this context, tasks for emotional face matching task and cognitive flexibility task were employed. Further information on the entire neuroimaging pilot is provided elsewhere [16]. Forty-seven children with mothers who displayed prenatal depressive symptoms (PDS) during pregnancy were invited for the current study. Control subjects (*n* = 37) were matched with the target population on age, gender, handedness and ethnicity and also invited to take part in the fMRI study. The Medical Ethical Committee of the Erasmus Medical Centre approved the study, and informed consent was obtained from a parent or a legal guardian prior to participation.

### Maternal depressive symptoms and child behavioral assessment

Maternal depressive symptoms were assessed using the Brief Symptoms Inventory (BSI) between 20 and 25 weeks of gestation and when the child was 3 years of age, referred to in the manuscript as prenatal depressive symptoms and postnatal depressive symptoms, respectively. This validated self-report questionnaire defines a spectrum of psychiatric symptoms, of which the six-item depression scale was used [18]. Mothers with scores higher than 0.75 display clinically relevant depressive symptoms, according to Dutch normative data [19, 20], which was used as a cutoff score. The control group had low BSI scores (0–0.67), compared to the PDS group (0.83–2.33). When children were around 6 years of age, parents completed the standardized Child Behavior Checklist 1.5–5 (CBCL; [21]). The CBCL for toddlers was used to obtain standardized parental reports of children’s internalizing and externalizing problems. This questionnaire contains 99 problem items, which are scored with regard to seven empirically based syndromes that were derived by factor analyses: emotionally reactive, anxious/depressed, somatic complaints, withdrawn, sleep problems, attention problems, and aggressive behavior. The summary internalizing scale is a summary score for items on the first four syndrome scales, and the externalizing scale is a summary score for attention problems and aggressive behavior. Each item is scored 0, 1 or 2 (0 = not true, 1 = somewhat or sometimes true, 2 = very true or often true) on the basis of the child’s behavior during the preceding 2 months. Higher scores indicated more problems. Good reliability and validity have been reported for the CBCL [22]. In the current study, the weighted sum scores of the internalizing and externalizing problem scales were used to check for potential behavioral differences between groups.

### Experimental paradigm

**Fig. 1:**
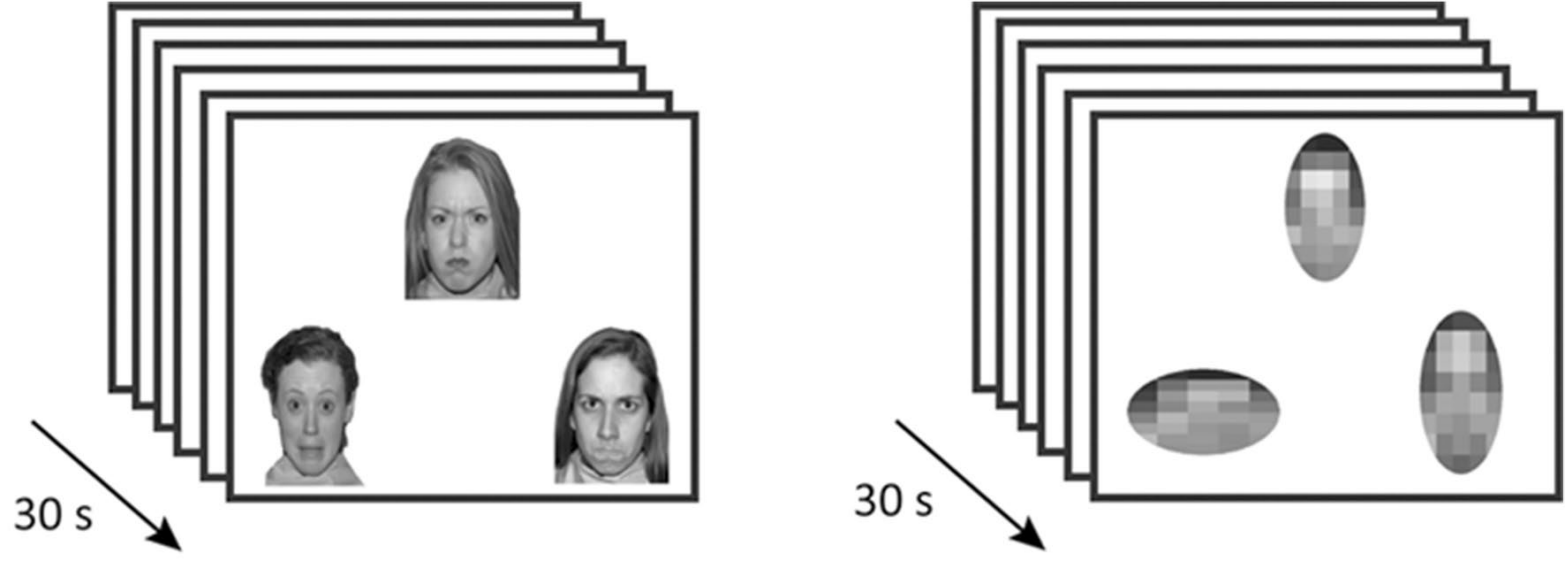
Overview of experimental task conditions. The task consisted of two emotional face blocks and three control blocks. Each block contained six trials of 5 s with a total time of 30 s per block. Participants were instructed to indicate which of the two items at the *bottom* matched the item at the *top*

Children performed an emotional face matching task (Fig. 1), consisting of two blocks of an emotion condition and three blocks of a control condition, which were presented in alternating order starting with a control block [23, 24]. Each block contained six trials of 5 s each. Three stimuli were presented simultaneously at each trail, either fearful and angry female face (emotion condition) or horizontally and vertically oriented ovals (control condition). Faces were of mixed ethnicities in line with our study population. For the emotion condition, children were instructed to identify the emotion of the cue face at the top of the screen and indicate which of the two target faces shown below matched in terms of emotional expression by pressing one of two buttons. Within the control condition, the geometric orientation of the ovals had to be matched. Two versions of the task were used equally often in each group, and response time and accuracy were monitored. Prior to the neuroimaging procedure, children were familiarized with the procedure in a mock scanner.

### MRI data acquisition

Scanning was carried out on a GE Discovery MR750 3 Tesla whole body MRI system (General Electric, Milwaukee, USA) using an 8-channel head coil as described previously [16]. Preceding the fMRI task, a structural scan was obtained. The high-resolution structural scan was acquired using a whole brain *T*_1_ inversion recovery fast spoiled gradient recalled (IF-FSPGR) sequence with the following parameters: TR = 10.3 ms, TE = 4, 2 ms, flip angle = 16°, matrix = 256 × 256, 186 contiguous sagittal slices with an isotropic voxel size of 0.9 mm^3^. Functional MRI was recorded using a gradient-echo blood oxygen level-dependent (BOLD) EPI sequence with a TR = 2000 ms, TE = 30 ms, flip angle = 85°, matrix × 64 × 64.

### fMRI behavioral data analysis

In five children, no behavioral data were recorded (control *n* = 3; PDS *n* = 2) due to a technical failure leaving us with thirty-four children that had correctly recorded behavioral data on task performance (response time and accuracy) (control *n* = 17; PDS *n* = 17). Additionally, datasets were excluded from behavioral analysis if children performed at chance level (control = 1, PDS = 2), giving us the final number used for analysis with 15 PDS children and 16 control children. Behavioral results were analyzed in SPSS 19.0 (SPSS, Chicago, Illinois, USA) using a repeated measures ANOVA, comparing between PDS children and controls. We did include these excluded children without recorded behavioral data, but with visual inspection of non-random engagement to the task at hand.

### Functional MRI data processing and statistical analysis

We were stringent by only including subjects with limited movement artifacts (less than 3 mm absolute movement for each direction vector; or any other related movement artifacts) leaving us with good quality data from 19 PDS children and 20 matched control children. Two children were included in the PDS group, who were exposed to prenatal maternal depressive symptoms based on self-report of the mother but for whom BSI scores were missing (Table 1). For the analysis of the functional scans, we used Statistical Parametric Mapping (SPM 8.0; http://www.fil.ion.ucl.ac.uk/spm). Groups were checked for differences in overall movement during scanning, assessed by independent t tests of mean scan-by-scan displacement for all six movement directions. Functional scans of participants were rigid body transformation aligned to their T1 image. The first four volumes were discarded to allow for T1 equilibration. Structural scans were segmented using an age appropriate (age of 7) tissue probability map toolbox (the Template-O-Matic (TOM8) [25]. The segmentation parameters were subsequently used to normalize functional and structural scans to Montreal Neurological Institute space (MNI 152) with the unified segmentation procedure implemented in SPM8. The normalized images were smoothed with a full width at half maximum kernel of 6 × 6 × 6 mm.

**Table 1:** Descriptive statistics of the participants

	Control	PDS	Statistics
Age [mean (SD)]	7.77 (0.95)	7.75 (0.76)	*F*(37;0.007), *p* = 0.95
Gender (% boys)	40	63	*X*2 = 1.6, *p* = 0.206
Ethnicity (%)			*X*2 = 0.219, *p* = 0.896
Dutch	70	63	
Non-Dutch, western	5	6	
Non-Dutch, non-western	25	31	
CBCL^a^ score at 6 years			
Internalizing [mean (SEM)]	7.9 (1.4)	9.3 (2.3)	*t*(37, −0.49), *p* = 0.623
Externalizing [mean (SEM)]	8.3 (1.5)	10.6 (2.4)	*t*(37, −0.24), *p* = 0.810
Prenatal BSI^b^ score [mean (SD)]	0.13 (0.22)	1.14 (0.4)	*U* = 340, *p* = 0.001

^a^Child Behavioral Checklist

^b^Brief Symptoms Inventory

Statistical analysis was performed using general linear models, in which the experimental blocks were modeled with 30-s boxcars based on condition (emotion or control) and convolved with the canonical hemodynamic response function. Seven covariates were included to remove any signal unrelated to the task (six movement parameters and whole brain mean signal of the functional scans). A one sample student *t* test was used to check voxel-wise for significant differences in the contrasts emotion > control. MarsBaR toolbox in SPM [26] and an anatomical amygdala mask from the Automated Anatomical Labeling Atlas (AAL) was utilized to extract overall contrast estimates for the bilateral amygdala. Univariate ANCOVA was used using SPSS to test for group differences in activity. We included gender as a covariate, because it was numerically imbalanced among the two groups. Results were robust also upon removal of influential values (more than 2.5 SD from mean).

To overcome missing values within the CBCL scales and the BSI postnatal values, multiple imputations were performed (Markov chain Monte Carlo; five imputations and ten iterations, using the prior CBCL assessment at 3 years of age, prenatal BSI assessments, gender, maternal characteristics and paternal emotional state as predictors). The imputed internalizing and externalizing scores were log transformed and were compared between groups using an independent student *t* test. Finally, a linear regression analysis was performed on the mean BOLD signal from the bilateral amygdala for the emotion > control contrast as dependent value with maternal PDS as predictor. To control for postnatal depressive symptoms of the mother, a second model included postnatal maternal depressive symptoms as predictor in addition to the PDS. In addition, a nonparametric spearman correlation analysis was performed to compare prenatal depressive symptoms and postnatal depressive symptoms with the BOLD signal from the bilateral amygdala for the emotion > control contrast as dependent value, to verify no bimodality was influencing our results.

## Results

Children of the PDS and control group did not differ significantly in age, ethnicity or gender (Table 1). Furthermore, groups did not differ significantly in externalizing or internalizing problems at age 6 years (Table 1).

As expected, the emotion condition was more challenging than the control condition, leading to slower responses [mean (SD) RT emotion condition = 2, 9 (0.5) s versus RT control condition = 1.7 (0.6) s]. *F*_Greenhouse–Geisser_(1, 29) (158; *p* < 0.001); and lower accuracy (mean percentage correct (SD) emotion condition = 81% (12%) compared to the control condition = 90% (12%) *F*_Greenhouse–Geisser_ (1, 29) (12.9, *p* = 0.001). Group identity did not affect overall accuracy [*F*_(1,29)_ = 0.22, *p* = 0.64] or response times [*F*_(1,29)_ = 0.41, *p* = 0.53]. Also, there were no significant group by condition interactions [accuracy *F*_(1,29)_ = 0.0, *p* = 0.99; reaction time *F*_(1,29)_ = 0.631, *p* = 0.43]. Thus, PDS and control children showed comparable task performance during fMRI, enabling us to compare neural responses between groups without evidence for potentially confounding behavioral differences.

**Fig. 2:**
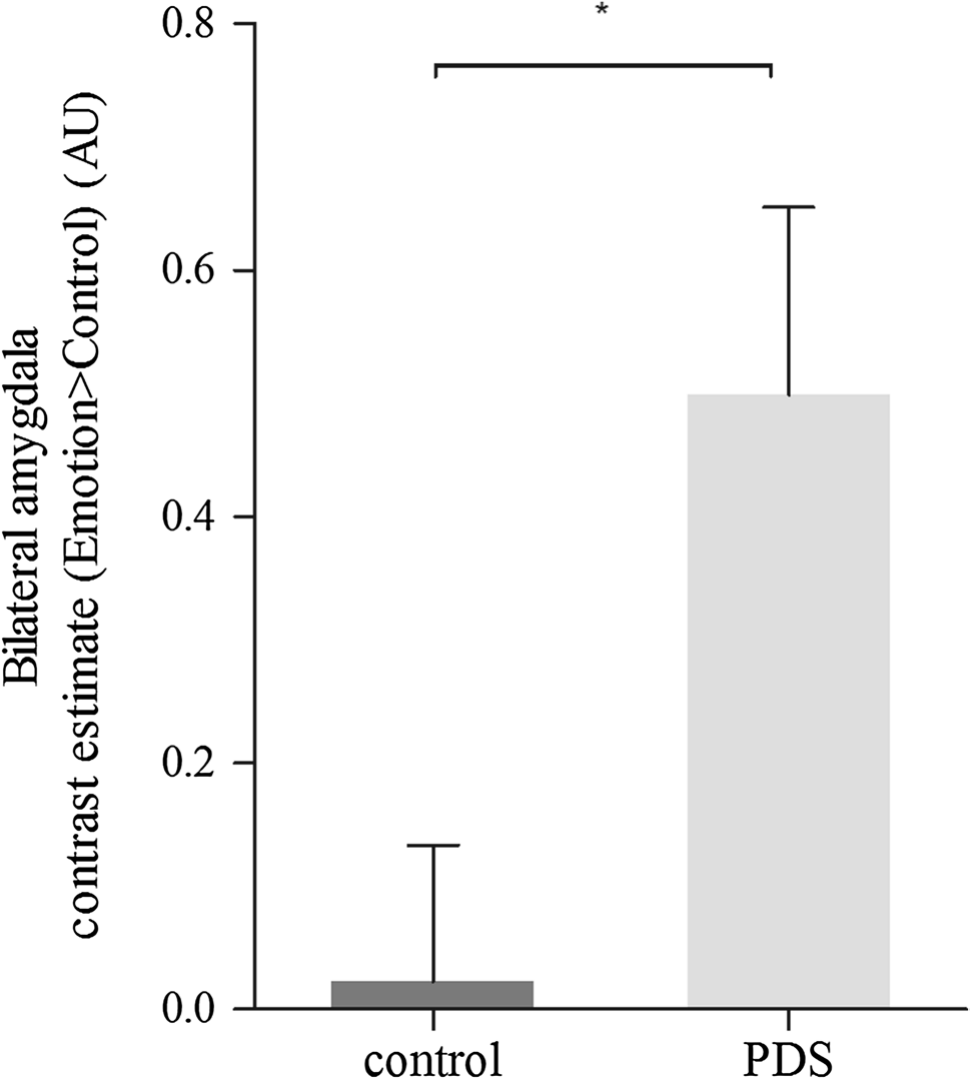
Amygdala hyperresponsiveness in children of mothers with clinically relevant prenatal depressive symptoms compared to control children. Displayed are mean BOLD fMRI contrast estimates extracted from the anatomically defined bilateral amygdala across all voxels for the contrast emotion > control condition separately for each group *F*(1,36) 7.02, *p* = 0.022 (*error bars* represent standard error of the mean, *AU* arbitrary units, *PDS* prenatal depressive symptoms, *p < 0.05)

In all children, emotional face matching resulted in significantly increased activity in the amygdala, hippocampus, fusiform gyrus and occipital lobe, compared to the control condition (Table 2). We extracted the mean bilateral amygdala BOLD signal in the emotion condition relative to the control condition for each group separately. Critically, the analysis showed that PDS children [mean (SD) = 0.49 (0.67)] had a stronger differential amygdala response [*F*(1,36) 7.02, *p* = 0.022; Fig. 2] compared to control children [mean (SD) = 0.02 (0.49)].

**Table 2:** Main effect of the emotional matching task

	Hemisphere	Number of voxels	Peak MNI coordinates			Peak *t* value
			*x*	*y*	*z*	
Emotion > control						
Fusiform gyrus	R	38	40	−50	−24	9.24***
Fusiform gyrus	L	61	−41	−50	−24	8.68***
Inferior occipital lobe	R	175	33	−87	−8	8.16***
Medial Temporal lobe	R	57	48	−36	0	9.54***
Inferior frontal gyrus	L	62	−56	20	20	9.89***
Inferior fontal gyrus	R	87	59	23	12	7.93***
Hippocampus and amygdala	L	15	−19	−10	−12	6.24**
Hippocampus	R	1	22	−32	0	5.54**
Lingual	L	9	−19	−91	−16	5.19**
Lingual	R	3	11	−32	−1	6.15**
Precentral lobe	L	4	−41	1	40	5.81**
Precentral lobe	R	1	40	5	60	5.79*
Precuneus	R	3	3	−61	32	5.64*

Depicted are the FWE corrected active regions for the emotion > control contrast *** *p* < 0.001 ** p < 0.01 * p = < 0.05

Linear regression analysis confirmed the positive relation between PDS and activity to emotional faces within the bilateral amygdala *B* = 38.5, 95% Confidence Interval (CI) 5.4–71.1, *p* = 0.023). Pre- and postnatal maternal depressive symptoms scores were positively correlated (*n* = 37; *r*_s_ = 0.63 *p* < 0.001), suggesting group differences were potentially driven by maternal depressive symptoms postnatally. However, when including maternal postnatal depressive symptoms within the regression model the positive relation between PDS and amygdala reactivity remained, although just missing formal significance (*B* = 43.7, 95% CI −1.2 to 88.5, *p* = 0.056). Interestingly, maternal postnatal depressive symptoms were not reliably associated with amygdala reactivity (*B* = −12.5, 95% CI −80.1 to 55.2, *p* = 0.717). Therefore, it does seem that prenatal depressive symptoms predict amygdala responsivity over and above any shared variance. Nonparametric correlation analysis confirmed this finding, prenatal depressive symptoms show a positive correlation with amygdala reactivity (*R*_s_ = 342 *p* = 0.038), while postnatal depressive symptoms did not (*R*_s_ = 0.236 *p* = 0.16).

## Discussion

We investigated the association between prenatal maternal depressive symptoms and brain activity in 6- to 9-year-old children. We provide initial evidence of an elevated amygdala response to negative emotional stimuli in children exposed to maternal depressive symptoms during pregnancy. While these findings by themselves do not permit firm conclusions on causality, they suggest that being exposed to maternal depressive symptoms prenatally may be related to enduring changes in later-life by affecting neural processing in young children.

An early and substantially larger study of the same prospective cohort, at a younger age, reported an association between prenatal maternal depressive symptoms and reduced head and body growth during fetal life and increased affective problems in early childhood [4, 20]. Prenatal maternal depression is also related to differences in the offspring’s physical health, social functioning and stress levels [10]. Here we show the association between PDS exposure and amygdala functionality in school-age children, at an age much younger than the typical age of onset of clinical relevant affective psychopathology. While we did not find any differences in internalizing or externalizing problems as a function of maternal depression, our sample was likely too small to reveal these effects [4]. To the best of our knowledge, altered amygdala functionality related to prenatal maternal depressive symptoms has not been investigated before. However, there are data on postnatal early life exposure to stressors, such as maltreatment or confinement to an orphanage on functional brain activity. These early life events are similarly related to higher amygdala reactivity to emotional stimuli, in both children and adults, corresponding to our current findings [27–29].

Pre- and postnatal maternal depressive symptoms are positively correlated [30]. Studies investigating the effects of pre- and postnatal exposure on psychopathology later in life have shown that prenatal exposure and postnatal exposure are differentially associated with family risk factors. Prenatal exposure to depression increases risk of depression at age 18 independent of exposure to maternal postnatal depression [9]. Although both prenatal maternal depression and postnatal maternal depression have been associated with altered amygdala structure in the offspring [12, 31], this previous study underlined that the two periods could influence child development in different ways [9]. In our study, adding maternal postnatal depressive symptoms, acquired at the age of 3 years postpartum, in the regression model resulted only in a slight attenuation of the association of PDS with amygdala reactivity. Indeed, postnatal depressive symptoms at this time point appeared not to impact amygdala reactivity. Seemingly, and perhaps surprisingly, prenatal rather than postnatal depressive symptoms of the mother are more strongly associated with amygdala hyperresponsivity, at least at the time points of our assessments. This would also suggest that the observed amygdala hyperresponsivity is not simply associated with an inherited vulnerability for depression [32] but related to an additional environmental cause within a critical prenatal period of development. It has to be noted, however, that we controlled for postnatal depressive symptoms only at the moment when the children were 3 years of age, meaning that transient changes in postnatal depressive symptoms, for instance immediately postpartum, are not accounted for.

The ability of humans to regulate their emotions is a key element of affective fitness. The amygdala is at the core of this regulatory process [6]. Failure to properly regulate emotional responses, as reflected in heightened amygdala reactivity to emotional stimuli, has been related to affective psychopathology or risk thereof [33, 34]. Children and young adults with affective disorders, for instance, show increased amygdala responsivity [35–37]. In addition, amygdala hyperactivity is associated with disease risk of psychopathology, even when individuals are currently in a disease-free state [38–40]. These findings indicate that amygdala hyperresponsivity could reflect a predisposition to psychopathology in later-life for this specific group of children, and follow-up studies would be necessary to confirm this line of thought.

Interestingly, in our study we found heightened amygdala responsivity to emotional stimuli in children exposed to PDS, whereas control children displayed a limited signal change in response to the same stimuli. In line with this, previous studies have shown an overall reduced responsivity of the amygdala among children, as compared to adolescents or adults, with the level of amygdala activity not discriminating between neutral and negative emotional stimuli [41–43]. Moreover, in a similar emotional face matching study in children, the healthy control group shows no significant amygdala activity increase associated with fearful faces compared to baseline [27]. Hence, it is interesting that we observed enhanced amygdala reactivity to negative emotional faces in the PDS exposed children at this young age, potentially suggesting a premature response compared to the general child population.

Limitations of our study have to be kept in mind. Most importantly, depression is a complex disease. Altered negative mood accompanies, for instance, dysregulation of the HPA-axis, altered immune function and negative lifestyle habits including smoking, increased alcohol intake, changes in food intake or sleep disturbances [44–46]. Therefore, prenatal maternal depression may expose the unborn child to a range of adverse conditions, but our sample size did not permit to elucidate their specific roles on amygdala hyperactivity. Secondly, we do not have specific parent–child interaction measures within this current sample, which could be of influence in brain development of the child [47]. Moreover, the emotional face matching task used in this study is tapping in the face processing capabilities of the young participants next to emotional processing. These two constructs are indistinguishable in our study, and future studies are necessary to separate the involvement of both aspects within the activation of the amygdala.

In sum, we provide initial empirical evidence that prenatal, clinically relevant depressive symptoms of mothers are associated with amygdala hyperresponsivity in their children at 6–9 years of age. Altered responsivity of the amygdala may serve as a risk factor for children’s mental health in later-life. Large-scale epidemiological long-term follow-up studies are necessary to confirm the link between prenatal maternal depression, amygdala functioning and risk of psychiatric disease in offspring. Such findings should trigger research into potential prevention strategies to counteract such long lasting consequences at an early stage. Our results are a first step forward in recognizing a mechanistic link between maternal mood during pregnancy, functional properties of the amygdala and later live vulnerability for psychopathology in offspring.
